# Supplementary material for: Are carbon emissions trading and green financial instruments synergistic? -Comprehensive quantitative research based on content analysis
Source: PLoS One. 2024 Mar 7;19(3):e0298601. doi: 10.1371/journal.pone.0298601 (PMC10919601; doi:10.1371/journal.pone.0298601)
Supplement: S1 Data — (ZIP) [file pone.0298601.s001.zip › Data/地级市Z_F工作报告.zip!/║╙─╧/╨┬╧τ╩╨/└┤╫╘╣1⁄2╓┌║┼í╛┬φ┐╦╩2╛▌═°í┐_2007.docx]

各位代表：

　　现在，我代表市人民政府向大会做工作报告，请予审议，并请各位政协委员提出意见。

　　一、2006年工作回顾

　　2006年，市人民政府在省委、省政府和市委领导下，在市人大、市政协监督支持下，团结带领全市人民，全面落实科学发展观，实施引资、项目双带动战略，优化经济结构，统筹城乡发展，促进社会和谐，较好完成了市十届人大三次会议确定的各项目标任务。初步核算（下同），全年地区生产总值达到633.4亿元，增长14.5%，其中，第一、二、三次产业分别增长8.9%、17.7%、12.2%；地方财政一般预算收入32亿元，增长26.1%；城镇居民人均可支配收入9455元，农民人均纯收入3653元，扣除价格因素，分别实际增长12.7%和16%。

　　一年来的主要工作是：

　　（一）强力推进招商引资和项目建设。招商引资工作取得新突破。香港景城、青岛双星、河北三鹿、黑龙江完达山等一批知名企业落户我市，新批外商投资企业53家，引进亿元以上项目54个；合同利用外资26673万美元，增长209.1%；实际利用外资13248万美元，增长120.2%；利用市外资金69.9亿元，增长45.6%。投资结构进一步优化，完成全社会固定资产投资425.2亿元，增长37.8%，工业投资占城镇以上投资比重为57.1%。实施千万元以上项目911个，同比增加264个，完成投资315.6亿元，增长40%，金龙集团年产6万吨精密铜管等47个重点项目竣工投产。千方百计解决项目所需的资金和土地问题，全年新增贷款绝对额和增幅均居全省第二；积极争取用地指标，基本保证了重点项目建设需要。努力改善投资环境，荣获“外商眼中河南最佳投资城市”称号。

　　（二）加大“三农”工作力度。积极推进社会主义新农村建设，全市用于改善农村生产生活条件的支出15亿元，增加4.1亿元。新建、改建农村道路1823公里，提前一年实现“村村通油路”目标，新增通班车行政村161个，通车率98.2%。新增农村沼气用户6.2万户，累计达31.7万户，入户率30.5%。新建改造“农家店”1996家。新解决农村15万人饮水安全问题。完成扶贫开发整村推进项目45个，当年2.8万人实现脱贫。实施农村劳动力转移培训14.4万人，完成劳务输出90.8万人、劳务收入39.8亿元。加快发展现代农业，全年粮食总产347.9万吨，增长12.3%，创历史新高；优质粮产量比重为82%，畜牧业产值占农业总产值比重为43%；新增无公害农产品认证42个、无公害畜产品产地认证17家。凤凰山森林公园超额完成年度植树任务，全市整体实现平原绿化高级达标。完成天然文岩渠清淤疏浚年度任务，开展小型农田水利设施“民办公助”试点，农田水利基本建设再夺省“红旗渠精神杯”。县域经济持续壮大，8个县（市）规模以上工业增加值占全市的60.9%，6个县（市）财政一般预算收入超亿元，其中辉县市和新乡县超过3亿元。

　　（三）调整优化产业结构。三次产业比例从上年的16.4：51.5：32.1调整为15.1：53：31.9；非公有制经济比重为58 %，提高3个百分点。高新技术产业增加值占工业增加值比重达到26%，高技术产业增加值比重达11%。工业利润显著提升，规模以上工业企业实现利润和利税分别增长111.1%、84.1%，创我市历史最好水平。完成进出口总额9.7亿美元，增长83.5%，其中出口6.3亿美元，增长80.4%，高新技术产品出口比重达80.2%。优势产业支撑作用增强，六大主导产业实现销售收入和利润分别占全市的63%、67%。全市主营业务收入超亿元的企业突破130户，其中金龙集团突破100亿元，新飞集团达到50亿元，并双双入选中国制造业500强。着力改造传统产业，6家重点造纸企业占整个行业产能的77.8%，水泥行业基本实现新型干法生产，纺织行业棉纱生产能力突破300万锭。加快淘汰落后生产能力，依法关闭44家水泥立窑企业和79条立窑生产线，全年单位生产总值能耗下降3%。园区经济和产业集群快速发展，5个省级以上园区共实施千万元以上项目114个，完成投资21.2亿元；制冷、电池、振动机械、医疗器械、起重装备、汽车及零配件六个产业集群初具规模。服务业发展提速。东方文化商业步行街等重点商贸项目竣工开业；完成社会消费品零售总额189.8亿元，增长15.5%；实现国内旅游收入18.5亿元、创汇165万美元，分别增长41.2%、32%；新开工商品房面积229.6万平方米，竣工面积163.9万平方米；邮电通信、金融业分别增长38.3%、10.4%。

　　（四）加快新乡都市区建设。37个产业聚集区完成基础设施和工业投资88.9亿元，入驻各类企业和项目453个，30分钟同城经济圈初步形成。新建、改造干线公路186公里，高速公路通车里程达192公里。小尚庄污水处理厂、生活垃圾无害化处理场正式运行；辉县市污水处理厂竣工调试，其他县（市）污水处理项目全部开工。完成热网工程主管道铺设任务。环境综合整治成效显著，基本完成市区主次干道整治和沟渠治理任务，市区58条小街巷面貌明显改观，新建公厕25座、改造公厕125座、建成垃圾中转站10座。城镇化率提高1.8个百分点。

　　（五）积极推进改革创新。列入省考核范围的国有企业当年完成或基本完成改制44户，累计完成或基本完成比例达96.4%。农村信用社增资扩股、国有粮食购销企业和市政公用行业改革改制基本完成。努力提高自主创新能力，新增省级工程研究中心3家、省级企业技术中心10家、博士后科研工作站3家。全年专利申请量1057件，居全省前列。科隆公司成为国家“863”计划重大项目课题承担单位，金龙集团“精密铜管四辊旋轧及四联拉关键技术研究”列入省“十一五”重大科技专项发展规划。

　　（六）全面发展各项社会事业。农村中小学现代远程教育工程提前竣工。新乡职业技术学院建设进展顺利，8个县（市）职业教育中心全部建成。新乡师专、平原大学、新乡教育学院“合并升本”通过全国高校设置评议委员会评审。潞简王墓进入世界文化遗产预备名录。改造乡镇卫生院4所，累计完成151所，占总数的97.4%。新建城镇社区服务中心20个、农村社区服务中心508个。加强计划生育工作，人口自然增长率为5.1‰。加大环保工作力度，四条省控河流出境断面水质达标率完成省定任务，市区空气质量优良以上天数316天。实现“全国质量兴市先进市”三连冠。新闻出版、广播电视、防震减灾、体育、审计、统计、史志、外事、侨务、档案、气象、测绘、人防等工作都取得了新的成绩。

　　（七）努力解决民生问题。城镇新增就业13.4万人，安置下岗失业人员6.5万人，其中“4050”人员2.5万人；发放再就业小额贷款8100万元；城镇登记失业率3.3 %；基本实现市区有劳动能力的“零就业”家庭至少有1人稳定就业。新建、改造24所乡镇敬老院，农村五保户集中供养率达到30.7%。提高城乡低保补差水平，21.1万城乡困难群众基本生活得到保障。为4.2万名农民工追讨工资2897.6万元。发放农村“两免一补”资金6297万元，资助学生56.1万人次；为城镇低保家庭发放“两免一补”资金52.9万元，资助学生4605人次。337.7万农民参加新型农村合作医疗，参合率89.1%；在全省率先开展全面的城市医疗救助，11.2万困难群众受益。开工建设经济适用房12.6万平方米，竣工10.8万平方米；为234户特困家庭发放廉租房补贴资金34.3万元。去年省、市两级向群众承诺办好的20件实事全部完成。

　　（八）切实加强精神文明和民主法制建设。深入开展社会主义荣辱观教育，思想道德建设取得新成绩。认真落实《行政许可法》，扎实推进依法治市，政府部门依法行政的能力与水平进一步提高。自觉接受人大及其常委会的法律监督、工作监督和政协的民主监督，严格执行人大及其常委会通过的决定和决议，人大代表议案、建议和政协委员提案办结率均为100%。狠抓安全生产工作，伤亡事故起数和死亡人数分别下降8%和8.2%。创新信访工作思路，千名干部下访活动效果显著。民族团结进步先进市创建活动取得明显进展。公安基层基础工作不断加强，严打整治斗争扎实有效，首次荣获全省“平安建设先进市”称号。坚持不懈地开展反腐败斗争，扎实推进机关效能建设，各级政府部门及工作人员的责任、法治和服务意识不断增强。

　　各位代表，过去的一年，我们在全面建设小康社会的道路上又迈出了坚实的一步，实现了“十一五”发展的良好开局。这是省委、省政府和市委正确领导的结果，是市人大、市政协积极支持的结果，是全市人民团结奋斗的结果。在此，我代表市人民政府，向各位人大代表和政协委员，向工作在全市各条战线上的广大工人、农民、知识分子、干部、驻新解放军指战员、武警官兵、公安干警及社会各界人士致以崇高的敬意！向关心、支持新乡建设的香港特别行政区同胞、澳门特别行政区同胞、台湾同胞、海外侨胞和国际友人表示衷心的感谢！

　　同时，我们也清醒地认识到，发展中还存在一些突出矛盾和问题。一是区域经济竞争力依然不强。我市的主要经济指标多数位于全省中游，在中原城市群9市中位次比较靠后。经济增长方式比较粗放，项目和产品的科技含量、附加值总体水平不高，结构调整和节能降耗任务依然很重。科技创新能力亟待加强，全市54户重点企业中拥有省级以上研发机构的仅23家，食品加工和水泥行业中没有省级以上研发机构。工业增加值、销售收入、利润、利税的总量偏小，绝对额分居全省第8位、第9位、第11位和第11位。农村社会事业投入不足，基础设施依然比较薄弱，多渠道增加农民收入的机制尚未建立，农民持续增收困难。二是解决民生问题的力度仍需加大。财政收支矛盾突出，公共产品和公共服务供给能力有待提高。就业和社会保障任务艰巨，特别是困难企业职工安置压力很大，养老保险基金欠缴严重，预计今年基金缺口将达到3.1亿元。群众看病贵、看病难问题还没有根本解决，食品药品安全不容乐观，卫河流域水污染治理任务还很艰巨，教育收费、安全生产、社会治安等关系群众切身利益的问题仍较突出。部分群众生活还比较困难，我市农村还有35.2万人生活在年收入944元的贫困线以下；城市还有11万困难人群月平均收入低于160元。三是政府自身建设还需进一步加强。有的政府部门和工作人员重管理轻服务，工作标准低，作风浮漂，效率低下；虚报浮夸、形式主义、官僚主义甚至滥用职权、腐败堕落等现象仍不同程度地存在，严重影响了政府公信力和发展环境。对此，我们将认真对待，切实解决。

　　二、2007年政府工作的总体要求和奋斗目标

　　2007年是实施“十一五”规划的重要一年，也是落实市九次党代会精神的开局之年。新的一年，我市经济社会发展面临着国家促进中部崛起、中原城市群核心区建设力度加大和国际国内产业转移步伐加快等诸多机遇。国家扩大内需的政策效应逐步显现，有利于我市以加工业为主的工业经济提速发展；中央提出又好又快发展的新思路，有利于我市优化经济结构，提高经济发展的质量和效益；坚持科学发展观和构建和谐社会的重大战略思想深入人心，有利于我市统筹协调各方利益，形成加快发展的强大合力。特别是经过全市上下的埋头苦干，我市已经具备了加快发展的基础条件，正处在乘势而上、跨越发展的关键阶段，只要我们始终保持敢为人先的创新精神、只争朝夕的进取意识和求真务实的工作作风，善于在国家宏观调控中抢抓机遇，勇于在激烈的区域竞争中迎接挑战，挖掘内在潜力，增创发展优势，就一定能够战胜任何艰难险阻，创造更加辉煌的业绩。

　　今年政府工作总的指导思想是：以邓小平理论和“三个代表”重要思想为指导，全面落实科学发展观，加快构建社会主义和谐社会，深入贯彻党的十六届六中全会和省八次党代会、市九次党代会精神，以工业化为主导，以效益新乡、创新新乡、生态新乡、和谐新乡建设为主线，以转变增长方式、提高经济效益为着力点，大力实施引资、项目双带动战略，整体推进社会主义新农村、县域经济、新乡都市区“三位一体”系统工程，进一步深化改革、加速开放，增强创新能力，提高行政效率，加快工业化、城镇化、农业现代化进程，着力改善民生，建设平安新乡，努力实现经济社会又好又快发展。

　　经济社会发展的奋斗目标是：地区生产总值增长12%以上，其中三次产业分别增长5%、15%和11%；全社会固定资产投资增长25%左右，其中工业投资增长30%；社会消费品零售总额增长13%；地方财政一般预算收入增长15%；城镇居民人均可支配收入增长10%，农民人均纯收入增长8%；城镇新增就业7万人，城镇登记失业率控制在4.5%以内；人口自然增长率控制在6‰以内；单位生产总值能耗降低4.5%左右；化学需氧量、二氧化硫排放量分别减少4%和8.3%。

　　 实现上述目标，任务十分艰巨。我们必须认真贯彻科学发展观，按照促进经济社会又好又快发展的总体要求，坚持发展这个第一要务，更加注重发展质量，多做一些加强薄弱环节、夯实发展基础、事关新乡长远的工作，多上一些环境污染少、资源消耗低、技术含量高的项目，努力实现速度、质量、效益相协调，人口、资源、环境相协调。坚持自主创新，更加注重增强科技创新能力，提高科学技术在经济增长中的贡献率，提高产业、产品市场竞争力。坚持以人为本，更加注重社会和谐，以更大精力解决人民群众最关心、最直接、最现实的利益问题，以更大力度推进社会事业发展和公共服务体系建设，让人民群众充分享受改革发展的成果。坚持深化改革，更加注重提高行政效率，坚决破除不利于又好又快发展的体制机制，认真解决影响发展环境的突出问题，充分激发全市上下加快发展的积极性和创造性，切实把经济社会发展转入科学发展的轨道。

　　三、加快经济转型步伐 努力实现区域经济又好又快发展

　　把国家宏观调控作为推进经济转型的重要契机，以建设效益新乡、创新新乡为主线，以转变经济增长方式、提高经济效益为着力点，力争在重点领域取得突破性进展。

　　（一）坚持引资、项目双带动战略，增强经济发展后劲

　　提高招商引资的层次和水平。进一步改善投资环境，提高办事效率和服务水平，增强对外商的吸引力。整合市直招商机构，完善招商引资考核评价和激励机制，加快招商引资信息平台建设。充分发挥各类工业园区和产业聚集区的招商引资主平台作用，积极推行产业链招商、委托招商、定向招商、以商招商等新型招商方式，切实抓好重点国家、重点区域、重点行业、重点企业的招商。组织参加香港投资贸易洽谈会、中博会等招商活动，筹办新加坡、墨尔本、上海、深圳、杭州等地新乡投资环境说明会。强化对招商项目的跟踪服务，确保香港景城中原软包装科技园、完达山乳制品加工等重大项目开工建设。力争新批外商投资企业55家，合同利用外资2亿美元，实际利用外资1.3亿美元；利用市外资金80亿元；进出口总额达到11.5亿美元。

　　加大项目谋划实施力度。根据国家产业政策导向，坚持新建与技改并重的原则，谋划实施一批成长前景好、产业链条长、拉动作用强、科技含量高的重大项目，保持固定资产投资较快增长。计划实施千万元以上项目672个，总投资990亿元，完成投资310亿元；其中重点项目146个，总投资518亿元，完成投资145亿元。加大赵固一矿年产240万吨原煤、新飞集团年产200万台冰箱生产线等100个续建项目投资强度，确保宝山2×60万千瓦火电、河南万向100万台汽车制动器及2万吨零部件等30个项目建成投产。开工建设新乡化纤年产4万吨高品质差别化粘胶短纤维、拓新公司年产1000吨核苷系列医药中间体等46个项目，完成年度投资53亿元。积极做好宝山电厂二期、宝泉抽水蓄能电站续建、卫华集团大吨位多功能起重机及重点煤化工项目的谋划争取工作。规范项目审批、核准及备案程序，禁止手续不完备和不符合国家产业政策的项目开工建设，提高新上项目质量。

　　努力解决项目建设中的资金、土地问题。抓住资本市场发展的新机遇，积极推进企业股票上市，力争5家企业实现上市或再融资。指导金融机构和企业建立长期稳定的新型合作关系，抓好担保、投资公司建设，力争全市新增贷款80亿元。正确处理经济发展与保护耕地的关系，加快推进“三项整治”，盘活存量土地资源，保证重点项目用地需要。高度重视集约节约利用土地，严格用地审批手续，建立完善园区土地利用评价体系，严格执行单位土地面积投资强度标准。大力推广标准化厂房建设，争取新开工20万平方米、竣工10万平方米。

　　（二）优化调整产业结构，全面提升产业效益和竞争力

　　大力发展现代农业。以高产、优质、高效、生态、安全的农畜产品为重点，进一步调整农业结构，畜牧业产值占农业总产值的比重达到45.5%。优化农业区域布局，以省农科院（原阳）现代农业科技实验示范基地为龙头，在原阳、长垣、延津、市郊四个重点区域规划建设现代农业示范中心。加强优质小麦生产、加工基地和种子基地及原阳大米、封丘金银花基地建设。推进沿黄绿色奶牛养殖带建设，开展“奶业发展年”活动，新增养殖小区28个，其中奶牛小区15个，总数分别达到300个和50个。完善动物疫病防控体系，确保不发生重大动物疫情。加快农业标准化建设，新建无公害农产品、绿色食品生产基地10万亩，新增无公害农产品和绿色食品认证15个、无公害畜产品产地认定企业12家。推进农业产业化经营，培育认定市级重点龙头企业20家、市级示范农民专业合作社30家。加快林业产业化进程，继续实施退耕还林、平原绿化和通道绿化工程。搞好大型灌区节水续建配套工程建设，开工建设南水北调工程新乡试验段，完成三郊口水库除险加固和天然文岩渠大车集至堰南河段清淤疏浚工程。

　　推进工业结构优化升级。以优势产业为支撑，以重点企业为龙头，以园区经济和产业集群为载体，优化产业结构，壮大产业规模，提升工业发展的质量和效益，走新型工业化道路。重点发展电子信息、生物与新医药、汽车及零配件、特色装备制造、食品、化工六大主导产业，力争年销售收入和利税增长30%以上。发展壮大高新技术产业，组织申报省级高新技术企业20家、高新技术产品30项，高新技术产业增加值、高技术产业增加值占工业增加值比重分别达到27%、11.5%。运用先进技术改造提升建材、造纸、纺织等传统产业。推动54户市重点企业提速发展，着力培育大型企业集团，力争12户省重点企业、3户高成长型企业年销售收入和利税平均增长35%以上，其他重点企业年销售收入和利税平均增长30%以上。大力发展园区经济，培育壮大产业集群，尽快理顺园区管理体制，加强软硬件建设，引导生产要素向优势企业、优势产业、优势区域集中，努力培育一批专业化分工明显、企业间协作紧密、产业链条完整、服务体系健全的产业集群，提高我市支柱产业整体竞争力。

　　提升服务业发展水平。以现代物流、商贸、文化、旅游、金融、信息、房地产、中介、社区、农业服务业等10大领域为重点，促进服务业业态升级和结构调整。加快大景城、沃尔玛等重点商贸项目建设，开工建设小店综合物流园区、华源电力仓储物流、河南长通物流数码港项目，争取开工建设桥北新区综合物流园区粮食仓储、新华粮食物流园区对外合作项目。加快旅游基础设施建设，重点发展南太行、宝泉、百泉、苍峪山、潞简王墓、比干林园等一批精品旅游景区，辉县市争取成功创建中国优秀旅游城市。在城乡社区积极发展社会救助与福利、未成年人教育、医疗卫生、便民利民、优抚助残、居家养老等服务项目，新建农村社区便民服务中心488个。

　　加大节能降耗工作力度。严格执行行业准入标准，淘汰落后工艺、设备和生产能力。推进循环经济试点工作，完善鼓励发展循环经济的政策措施，积极开发和推广节能新技术、新工艺和新产品，抓好重点领域、重点行业、重点企业的节能降耗和余热余压利用、能量系统优化、绿色照明等节能项目。大力发展节能建筑，新增居住建筑要达到节能65%的设计标准。

　　（三）推进“三位一体”系统工程，加快城乡一体化步伐

　　积极建设社会主义新农村。进一步加大对农村基础设施和社会事业的资金投入，努力解决农村群众最关心、要求最迫切的生产生活问题。新建、改造农村道路500公里，通油路的村全部通班车；新增沼气用户6万户；确保再解决农村17万人饮水安全问题；年内实现村村通光缆，电话入户率达到85%以上，完成207个自然村“广播电视村村通”工程；每个行政村建成一个村级组织活动场所；建设“万村千乡”工程配送中心2个，新建和改造“农家店”1010个。多渠道增加农民收入，做好对种粮农民的直接补贴、良种补贴、农机具购置补贴、农业生产资料综合补贴和重大农业技术推广专项补贴，努力增加种养业、林业收入，积极发展农村二、三产业，加大劳务输出力度，完成农村劳动力转移就业引导性培训20万人、职业技能培训10万人，实现农村劳动力转移就业100万人、劳务收入54亿元。抓好社会主义新农村“一县一镇百村”示范点建设，实现垃圾处理规范化，确保60%的示范村通自来水，村内道路实现硬化。加大对革命老区、贫困地区、少数民族聚集地区的扶贫开发力度，加快整村脱贫步伐。

　　发展壮大县域经济。坚持“工业兴县”战略，完善促进县域经济发展的政策体系，大力发展民营经济，培育特色产业，促进产业集群发展。推进37个产业聚集区的起步区建设，基本完成路网、电网等八项基础设施建设任务，重点培育以黄河三桥、107新线及胡韦线为轴线的新郑产业发展轴。扶持纳入国家比照西部大开发政策支持的封丘县、原阳县加快发展。力争县域完成生产总值490亿元，增长15%；完成财政一般预算收入18.5亿元，增长20%。

　　全面推进新乡都市区建设。编制完成新一轮城市总体规划、村镇建设规划、中心村规划、“城中村”改造规划和水系生态环境规划。建立城乡一体的规划管理和执法体系，扩展规划管理的深度和广度，确保城市规划审批执行率100%。完善市域路网体系，济东高速公路新乡段年内建成通车，争取开工建设安新高速公路改扩建工程。新建改建干线公路184公里、城市道路7条共12.5公里。综合整治小街巷50条。改造提升300座公厕，其中旱厕200座。开工建设客运东站和货运东站。高起点规划建设桥北新区，积极推进河南科技城等重大项目，使之成为新乡与郑州城市功能和产业对接的桥头堡。加快新区核心区建设，开工兴建平原博物院等大型公共建筑工程。完善鼓励农民进城的优惠政策，城镇化率提高1.7个百分点。

　　（四）提升自主创新能力，强化科技创新的支撑作用

　　建立健全区域自主创新体系。市财政安排科学技术专项资金1371万元，比去年增加427万元。进一步加大对企业科技创新的扶持力度，提高各类科技扶持引导资金的使用效率。引导、鼓励企业加强与科研院所、高等院校的研发合作，逐步建立以高等院校、科研院所、重点实验室为依托的知识创新平台和以工程（技术）研究中心、企业技术中心等为依托的技术创新平台。新组建市级企业研发中心20家，组织申报国家级企业技术中心1家、省级企业技术中心5家、省级工程（技术）研究中心5家。组织申报省级以上科技计划项目30项，推荐30个项目参加省科技进步奖评审，努力在新材料、新能源、生物医药、电子信息等重点领域取得新突破。

　　营造良好的创新环境。强化企业的创新主体地位，引导企业加大研发投入。搭建以企业需求为导向的科技服务平台，建设科技成果信息交流中心，建立支持企业和企业家创新的服务体系和援助机制。加大知识产权保护力度，维护企业和专利权人的合法权益。大力引进和培养创新型人才，健全激励机制，为科技人员实现创新提供良好的外部环境，营造尊重和珍惜人才、有利于创新人才脱颖而出的浓厚氛围。

实施名牌强市和专利兴市战略。加强对名牌产品的培育和保护，着力培育一批市场占有率高、效益好的名牌产品，争创中国名牌产品2个、省名牌产品18个、省优质产品10个、国家免检产品2个、驰名商标2个、著名商标9个。引导企业积极参与标准化工作，提升标准化水平。培育知识产权优势区域和优势企业，积极申报国家专利试点城市，全年申请专利1000件以上，其中发明专利比例达到15%。

　　（五）坚定不移地推进各项改革，增强发展驱动力

　　推进国有企业改革。加大对已改制企业的跟踪服务力度，帮助其解决改制遗留问题，完善公司法人治理结构，建立现代企业制度。稳妥推进市属特困企业改制，力争基本完成局委属企业改制任务。全面实行国有资产经营目标责任制，规范企业产权交易行为，防止国有资产流失。

　　推进财税、金融、投资体制改革。深化县乡财政体制改革。进一步加强财政监督，推行“以票控费”管理改革，规范非税收入收缴和监管。深化政府采购改革，有效降低行政成本。依法加强税收征管，推行发票税控。积极推进市商业银行向现代股份制商业银行转变。全面完成农村信用联社统一法人工作。深化投资体制改革，进一步落实企业投资自主权，完善企业投资项目核准制和备案制；出台新乡市《政府投资项目管理办法》和《政府投资项目代建制管理办法》，加强政府投资管理。

　　推进其他各项改革。巩固、完善和深化乡镇机构改革，稳妥推进事业单位改革。全面推行“编制审核”制度，建立机构编制管理与财政预算管理配套协调的约束机制。加快征地制度改革，建立健全合理的征地补偿机制。全面完成非国有粮食购销企业改制任务。

　　四、高度关注民生 努力建设社会主义和谐社会

　　始终把关注民生作为政府工作的重中之重，以和谐新乡、生态新乡建设为主线，以增加群众收入、改善人民生活为着力点，以办好今年省、市两级已公布的关注民生20件实事为抓手，全面发展各项社会事业，统筹协调各方利益关系，认真解决涉及群众切身利益的矛盾和问题，使全市人民共享改革发展的成果和实惠。

　　（一）扎实做好就业和社会保障工作。千方百计增加就业。积极开发公益岗位，拓宽就业渠道，逐步建立城乡一体的劳动力市场，重点做好下岗失业人员和关闭破产企业人员再就业工作。筹资8000万元，为下岗失业人员再就业提供社会保险补贴、岗位补贴，完成再就业培训2.5万人。为2400名下岗失业人员进行贷款担保，发放再就业小额贷款7100万元。力争城镇新增就业7万人，下岗失业人员再就业2.4万人，其中“4050”人员再就业8800人。建立健全社会保障体系。扩大社会保险覆盖面，加大养老保险费征缴力度，推进失业保险市级统筹，建立失地农民基本养老保险制度，完善城乡社会救助体系。规范城市低保管理，做到应保尽保。农村低保对象增加至12万人，低保补差标准由20元提高到30元。加快社会福利社会化步伐，推进惠民院和福利园区建设，力争全市社会养老床位达到4900张。新建10所、改造10所乡镇敬老院。着力解决困难群体住房难问题。市区新开工经济适用房15.7万平方米，竣工12万平方米，解决1300户困难群众的住房问题；新增加100户人均住房建筑面积10平方米以下的特困家庭享受廉租住房补贴。

　　（二）优先发展教育事业。推进义务教育均衡发展。落实农村义务教育经费保障机制，新机制所需资金全部纳入财政预算，新增教育经费主要用于农村教育。加强“两基”薄弱环节建设，确保顺利通过国家检验。加快推进中小学布局调整，制定并启动市区学校布局调整专项规划，合并农村中小学80所。加强农村中小学校舍维修改造工作，更新农村中小学课桌凳4万套。加大教师资源统筹使用力度，强化薄弱学校师资培训，实现义务教育资源的合理配置。保证困难弱势群体学生接受义务教育的权利，全部免除农村义务教育阶段学生学杂费，继续对农村困难家庭子女免费提供教科书并补助寄宿生生活费。认真解决农民工同住子女义务教育阶段上学难问题，做到应入尽入、待遇平等。继续对城市低保家庭子女上学实施“两免一补”。认真执行义务教育学校“一费制”，有效制止教育乱收费。逐步普及高中教育，新建、改造一批普通高中，市区高中阶段入学率达到85％以上，努力提高农村高中阶段入学率。大力发展职业教育，积极推进职业教育各项改革，建立行业、企业、学校共同参与的办学机制。扩大中等职业学校办学规模，加快新乡职业技术学院建设，启动平原职业技术学院项目，开展国家级和省级示范性职业学校创建工作。努力为驻市高校排忧解难，支持高等院校加快发展。经过几年努力，争取建成一批国家级或省级高素质人才培训基地。

　　（三）加快发展医疗卫生事业。加强医疗卫生基层基础建设，缓解城乡居民看病贵、看病难问题。增加县级医院、乡镇卫生院基本医疗设备配置，完成剩余3所乡镇卫生院改造任务。新建、改建1000所标准化农村卫生室。转型改建21个城市社区卫生服务中心和69个城市社区卫生服务站，覆盖市区90％以上居民，对社区内60岁以上的居民逐步建立健康档案。进一步加强疾病防控体系建设，提高突发公共卫生事件应急处置速度和效能，做好艾滋病、结核病等传染病的预防和控制。完成市、县两级卫生监督机构改革，整顿和规范医疗服务秩序。扩大单病种最高限价病种数量，对非营利性医疗服务机构全部实行药品、高值耗材集中招标采购，下决心降低药品价格和医疗收费。完善城市医疗救助体系，重点加强大病医疗救助和医前救助。资助农村困难群众参加新型农村合作医疗，确保市辖区农民全部纳入参合范围，实现全区域覆盖。

　　（四）大力发展文化事业和其他社会事业。努力建设文化强市。建成农村文化大院180个，组织开展好争创全省文化先进县、乡活动。依托城市社区和农村文化大院，发展广场文化、节庆文化、民间文化。做好潞简王墓申报世界文化遗产工作。完成市直艺术团体改革。推进新乡广电网与省网整合。着力发展文化旅游业、印刷包装业和传统工艺品制作业，形成“三业鼎立”的新乡文化产业特色。促进各项社会事业均衡发展。稳定低生育水平，加强出生人口性别比问题综合治理。依法保护妇女、未成年人、残疾人和老龄人的合法权益，建设县级青少年活动中心，创建爱老模范城。认真做好新闻出版、广播电视、防震减灾、体育、审计、统计、史志、外事、侨务、档案、气象、测绘、人防等工作。

　　（五）加强环境保护和生态建设。加大污染防治力度，重点整治地表水污染和局部区域大气污染。痛下决心抓好卫河流域水污染治理，建立科学的监测体系，加强对重点行业和企业的排污监管力度，对卫河流域涉水企业和项目实施深度治理，确保出境断面水质达到省政府要求，确保我市饮用水水源地水质100%稳定达标。拆除市区供热管网覆盖区域内的采暖锅炉，所有燃煤电厂实现烟气脱硫治理。争取市医疗垃圾无害化处理场投入运行。加大污水处理费征收力度，所有县（市）污水处理厂和垃圾处理场建成投运。构建环城生态体系。完成凤凰山森林公园年度造林绿化任务。实施县级环城生态防护林规划，抓好“三圈四层次”林业生态建设。推进卫源湖、贾太湖、牧野湖、东湖、凤凰山湖的建设及绿化，实施人民胜利渠清挖整治、卫河市区段景观建设等水系景观配套工程。做好国家园林城市、森林城市、文明城市、环保模范城市的建设和申报工作。

　　（六）加强精神文明和民主法制建设。弘扬“厚善、崇文、敬业、图强”的新乡城市精神，健全社会信用体系，深化社会主义荣辱观教育，抓好社会公德、职业道德和家庭美德建设。认真贯彻《人大常委会监督法》，自觉接受市人大及其常委会的法律监督、工作监督和市政协的民主监督，主动接受社会公众监督和舆论监督，认真办理人大代表议案、建议和政协委员提案。广泛听取各民主党派、工商联、无党派人士的意见和建议，发挥市工会、共青团、妇联、科协、文联、侨联、社科联、残联等人民团体的桥梁纽带作用。加强社区建设，完善基础设施，增强服务功能，开展和谐社区创建活动，提高基层社会管理水平。大力支持国防建设，做好优抚和退伍安置工作，加强民兵预备役队伍建设，力争实现全国双拥模范城“四连冠”。确保食品药品安全，继续实施食品放心工程，大力开展食品安全专项整治，加强对药品生产、流通等环节的监管力度，建立健全覆盖城乡的食品药品监管网络。加强安全生产工作，严格落实安全生产两个责任制，强化安全生产基层基础工作，健全应急救援机制，有效遏止重大安全事故发生。继续做好民族宗教工作，深入开展民族团结进步先进市创建活动。贯彻落实《信访条例》，坚持以群众工作统揽信访工作，实现信访工作由中间环节批转向源头防范和矛盾化解转变。加强社会治安综合治理，强化公安、司法基层基础工作，加大“严打”力度，维护社会稳定，建设平安新乡。

　　五、切实加强责任、法治、服务型政府建设

　　以提升行政效率为核心，以增强政府的公信力和执行力为出发点，切实转变政府职能，改进工作作风，全面提高科学行政、民主行政、依法行政水平和服务水平。

　　切实转变政府职能。加快推进政企、政事、政资、政府与市场中介组织分开，充分发挥市场配置资源的基础性作用，进一步强化政府的社会管理和公共服务职能。优化公共资源配置，调整财政支出结构，加强公共设施建设，更多地向农村、基层和贫困地区倾斜，向社会建设和公共服务领域倾斜，逐步建立覆盖城乡的基本公共服务体系。认真贯彻实施《行政许可法》和《公务员法》，全面推进依法行政，严格按照法定权限和程序办事，保证政府行为合法适当。健全政务公开的工作机制，加快电子政务和政府门户网站建设，认真办好政府公报、市长电话、市长信箱和行风热线，重点关注群众普遍关心和涉及切身利益的问题，畅通群众反映诉求的通道，保障人民群众的知情权、参与权和监督权。完善政府重大事项社会公示、听证制度，建立健全了解民情、反映民意、集中民智的政府决策机制，推进决策民主化和科学化。

　　全面提升行政效率。以“行政效率提速年”活动为载体，进一步深化行政审批制度改革，强化行政服务中心的职能作用，严格实行“一个窗口”对外，严格落实服务承诺制和限时办结制，健全联审会办运作机制和外来投资全程代理服务运作机制，完善电子政务，推行网上审批。规范行政权力运行，加强对行政权力运行的监督，狠抓机关规范化管理，实行市直机关科室（机构）季评排序和效能告诫制度，建立各级政府、职能部门之间以及企业、投资商对政府服务的客观评价机制，健全以绩效考核、效能监察和行政问责为核心的行政监督机制，强化责任、有序、高效的工作推进机制，完善以效率为核心的优化环境长效机制，切实解决好基层和群众反映强烈的突出问题，严肃整治行政不作为和乱作为，严厉查处破坏发展环境、损害新乡形象的人和事，确保各级行政机关规范、协调、高效、廉洁运转。

　　大力加强作风建设。政府各级各部门和全体公务人员一定要坚持讲大局、讲原则、讲纪律，始终站在全局的利益和高度上思考问题、认识问题、解决问题，严禁推诿扯皮、各自为政，严禁虚报浮夸、搞形象工程，严禁工作棚架、有令不行。一定要坚持高标准、严要求，讲实话、办实事、求实效，敢于触及矛盾，勇于承担责任，善于创造性地开展工作，积极主动地解决好各种热点难点问题。一定要牢记我们是人民的公仆，任何时候都要视人民的利益高于一切、重于一切、先于一切，任何时候都要把困难弱势群体的生活放在心上，经常深入社会最底层，倾听群众呼声，了解群众心愿，切身体验基层群众的真实生活，多做雪中送炭的事情，不遗余力地解决好他们最关心、最直接、最现实的利益问题，绝不能高居庙堂之上，漠视民间疾苦。要高度关注基层干部的工作和生活，帮助他们解决实际困难，解除后顾之忧。要进一步精简会议和文件，改进会风和文风，严格控制各类达标评比活动。要切实加强廉政建设和反腐败斗争，重点治理和纠正损害群众利益的部门和行业不正之风，严厉查办滥用职权、贪污贿赂、官商勾结、权钱交易等违纪违法案件。要始终牢记“两个务必”，坚持勤俭节约，严格控制公务消费，坚决刹住讲排场、比阔气、铺张浪费等不良风气。

　　各位代表！新的一年，各项任务艰巨繁重，我们深感责任重大。让我们紧密团结在以胡锦涛同志为总书记的党中央周围，高举邓小平理论和“三个代表”重要思想伟大旗帜，在市委的坚强领导下，认真落实科学发展观，全面建设和谐社会，奋力开创新乡经济社会又好又快发展的新局面！
